# Editorial for Special Issue: “Molecular Mechanisms Underlying Fatty Liver Disease: From Pathogenesis to Treatment”

**DOI:** 10.3390/cimb47030160

**Published:** 2025-02-27

**Authors:** Haibo Dong

**Affiliations:** Center for Translational Biomedical Research, University of North Carolina at Greensboro, North Carolina Research Campus, Kannapolis, NC 28081, USA; h_dong@uncg.edu

Metabolic-dysfunction-associated fatty liver disease (MAFLD) and alcohol-associated liver disease (ALD) represent two of the most prevalent chronic liver diseases globally, collectively affecting hundreds of millions of individuals. MAFLD, formerly known as non-alcoholic fatty liver disease (NAFLD), is closely associated with metabolic syndrome, obesity, and type 2 diabetes [1]. The global prevalence of MAFLD has surged from 25.3% (1990–2006) to 38.2% (2016–2019), marking a nearly 50% increase over the past three decades [2]. This rise has positioned MAFLD as the leading cause of chronic liver disease worldwide. The escalating prevalence is largely driven by the widespread adoption of unhealthy dietary patterns characterized by high intake of saturated fats, refined sugars, and processed foods [3,4].

In contrast, ALD is primarily attributed to excessive alcohol consumption. Harmful alcohol use is responsible for over 3 million deaths annually, with liver cirrhosis accounting for a significant proportion of these fatalities [5,6]. Although MAFLD and ALD have distinct etiological drivers, they frequently coexist in clinical settings, as the Western diet and alcohol consumption are often intertwined [7,8]. This overlap not only exacerbates the liver’s susceptibility to damage but also accelerates disease progression, creating a synergistic detrimental effect on liver health. 

While MAFLD and ALD share common pathological features, such as hepatic steatosis, oxidative stress, and inflammation, their underlying mechanisms differ significantly. MAFLD is primarily driven by metabolic dysfunction, including insulin resistance, dyslipidemia, and systemic inflammation [9]. Conversely, ALD is characterized by direct alcohol-induced hepatotoxicity, oxidative stress resulting from alcohol metabolism, and immune dysregulation [10,11]. These distinct pathological processes present unique therapeutic challenges and opportunities.

Understanding the differences and the interplay between these two conditions is essential for developing more effective and personalized therapeutic strategies. By addressing their shared features while accounting for their unique pathologies, we can make significant strides in alleviating the burden of these increasingly prevalent liver diseases.

This Special Issue of *Current Issues in Molecular Biology* brings together eight impactful studies that address critical aspects of MAFLD (NAFLD) and ALD, focusing on their cellular and molecular mechanisms while also exploring innovative therapeutic interventions. For example:

The central role of insulin resistance in the progression of MAFLD is a recurring theme across several studies. Vesković et al. [12] shed light on how insulin resistance triggers hepatic triglyceride accumulation through pathways involving oxidative stress, ER stress, and lipotoxicity. Their review also highlighted the interplay between circadian rhythm disruptions and metabolic dysfunction, proposing clock genes as novel therapeutic targets. This liver-centric perspective establishes a solid foundation for addressing MAFLD pathogenesis.

Complementing this, Tao et al. [13] examined the gut–liver axis as a key regulator of liver health, particularly in the context of drug-induced liver injury (DILI). They demonstrated how disruptions in gut microbiota composition and bile acid metabolism can exacerbate liver damage, emphasizing the therapeutic potential of probiotics, bile acid modulators, and fecal microbiota transplantation. These findings resonate with the broader understanding of how gut–liver interactions shape the progression of liver diseases.

Further extending the discussion on gut microbiota, Martin-Grau and Monleón [14] explored the role of microbiota-related metabolites, such as SCFAs, bile acids, and branched-chain amino acids, in MAFLD. These metabolites were identified as critical mediators of inflammation and lipid metabolism, offering potential biomarkers and therapeutic targets. Their work reinforces the significance of microbiota modulation in tackling metabolic liver diseases.

As the search for effective treatments continues, Lee et al. [15] introduced warm acupuncture (WA) as a promising non-pharmacological intervention for MAFLD. Their study demonstrated that WA activates the AMPK/SREBP1/ACC pathway, reducing lipid accumulation and improving liver histology. The findings suggest that acupuncture could complement existing therapeutic approaches, aligning with efforts to explore alternative therapies for liver health.

On the molecular front, Kwon et al. [16] identified dimethyloxalylglycine (DMOG) as an inhibitor of lipogenesis, acting through the suppression of SREBP1c independently of the hypoxia-inducible factor-1-alpha (HIF1A). By suppressing the transcription and expression of SREBP1c and its downstream lipogenic genes, including FASN, ACACA, and SCD1, DMOG offers a promising new approach for targeting hepatic lipid metabolism in fatty liver diseases. Interestingly, their findings also suggest potential redundancy in the HIF1A and INSIG2 pathways, shedding light on the complexity of SREBP1c regulation. This novel mechanism offers a pathway to target lipid metabolism in fatty liver diseases, providing new avenues for therapeutic development.

Adding a layer of complexity, Nair and Weiskirchen [17] shifted the focus to liver tissue engineering (LTE) as a complementary or alternative approach to liver transplantation. Their review discussed the challenges of donor shortages, immune rejection, and post-transplant complications, proposing LTE as a solution to these bottlenecks. They highlighted advances in whole-organ bioengineering, recellularization strategies, and the integration of biomaterials for liver regeneration. The potential of stem cells, decellularized scaffolds, and 3D bioprinting to create functional liver tissue for drug testing and transplantation was particularly noteworthy.

Together, these contributions weave a rich narrative of interconnected mechanisms underpinning MAFLD (NAFLD), ALD, and related conditions. From the pivotal role of insulin resistance and the gut–liver axis to innovative therapeutic interventions like WA and DMOG, this Special Issue underscores the necessity of multidisciplinary approaches to tackle the global burden of liver disease.

We are immensely grateful to all the authors for their invaluable contributions and to our reviewers for their rigorous evaluations. We hope this collection inspires further research and facilitates the development of transformative solutions for liver disease management.
